# Genetic association analysis identifies a role for *ANO5* in prostate cancer progression

**DOI:** 10.1002/cam4.2909

**Published:** 2020-02-06

**Authors:** Chia‐Cheng Yu, Lih‐Chyang Chen, Chao‐Yuan Huang, Victor C. Lin, Te‐Ling Lu, Cheng‐Hsueh Lee, Shu‐Pin Huang, Bo‐Ying Bao

**Affiliations:** ^1^ Division of Urology/Transplant Surgery Department of Surgery Kaohsiung Veterans General Hospital Kaohsiung Taiwan; ^2^ Department of Urology School of Medicine National Yang‐Ming University Taipei Taiwan; ^3^ Department of Pharmacy College of Pharmacy and Health Care Tajen University Pingtung Taiwan; ^4^ Department of Medicine Mackay Medical College New Taipei City Taiwan; ^5^ Department of Urology National Taiwan University Hospital College of Medicine National Taiwan University Taipei Taiwan; ^6^ Department of Urology E‐Da Hospital Kaohsiung Taiwan; ^7^ School of Medicine for International Students I‐Shou University Kaohsiung Taiwan; ^8^ Department of Pharmacy China Medical University Taichung Taiwan; ^9^ Department of Urology Kaohsiung Medical University Hospital Kaohsiung Taiwan; ^10^ Graduate Institute of Medicine College of Medicine Kaohsiung Medical University Kaohsiung Taiwan; ^11^ Department of Urology Faculty of Medicine College of Medicine Kaohsiung Medical University Kaohsiung Taiwan; ^12^ Center for Cancer Research Kaohsiung Medical University Kaohsiung Taiwan; ^13^ Sex Hormone Research Center China Medical University Hospital Taichung Taiwan; ^14^ Department of Nursing Asia University Taichung Taiwan

**Keywords:** anoctamin, biomarker, prognosis, progression, prostate cancer

## Abstract

Anoctamins were originally identified as a family of calcium‐activated chloride channels, but recently their roles in the development of different types of malignancies were suggested. Here, we evaluated the associations between 211 common single‐nucleotide polymorphisms in 10 anoctamin genes with biochemical recurrence (BCR) after radical prostatectomy (RP) for localized prostate cancer. Four SNPs (*ANO4* rs585335, *AN* *O5* rs4622263, *ANO7* rs62187431, and *ANO10* rs118005571) remained significantly associated with BCR after multiple test correction (*P* < .05 and *q* = 0.232) and adjustment for known prognostic factors. Expression quantitative trait loci analysis found that *ANO5* rs4622263 C and *ANO10* rs118005571 C alleles were associated with decreased mRNA expression levels. Moreover, lower expression of *ANO5* was correlated with more advanced tumors and poorer outcomes in two independent prostate cancer cohorts. Taken together, *ANO5* rs4622263 was associated with BCR, and *ANO5* gene expression was correlated with patient prognosis, suggesting a pivotal role for *ANO5* in prostate cancer progression.

## Graphical abstract

Our genetic association study identified that *ANO5* rs4622263 was associated with recurrence‐free survival in patients with prostate cancer following radical prostatectomy. Additional analyses also provided evidence that rs4622263 affects *ANO5* expression, which in turn is correlated with tumor aggressiveness and patient prognosis.

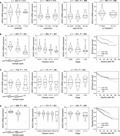

## 1 INTRODUCTION

Prostate cancer is one of the most common cancers affecting men worldwide. Most patients have clinically localized prostate cancer at the time of diagnosis but some develop aggressive prostate cancer, eventually leading to death. Radical prostatectomy (RP) is a widely used treatment for localized prostate cancer with excellent control; however, 20%‐40% of patients still experience biochemical recurrence (BCR) within 10 years of RP.^1^, ^2^ Although we currently have several predictors (prostate‐specific antigen [PSA], Gleason score, and cancer stage) to risk stratify patients with prostate cancer, their clinical outcomes can vary widely. Identifying additional prognostic factors could further improve the management of personalized treatment for these patients.

Anoctamins, also known as transmembrane 16 proteins, are a family of calcium‐activated chloride channels that comprise 10 members (ANO1‐10). Anoctamins play important roles in regulating membrane excitability, ion homeostasis, and cell volume during endo/exocytosis, as well as in cell proliferation.^3^ Recent studies suggest that some anoctamins may be relevant to the proliferation and progression of a number of cancers, including gastrointestinal stromal tumors, breast cancer, and prostate cancer.^4^, ^5^, ^6^ Although the detailed mechanisms by which anoctamins regulate tumorigenesis are still unclear, it has been speculated that they can transiently increase intracellular calcium levels, which could activate the Ras/Raf/MEK/ERK signaling pathway to affect cell proliferation.^7^ Considering this, it is important to comprehensively investigate the relationship between anoctamins and clinical prostate cancer outcomes.

In a recent Nordic Twin Study of Cancer, genetic factors were estimated to account for 57% of the variation in prostate cancer risk,^8^ suggesting the existence of hereditary factors that influence prostate cancer initiation. However, only a few studies explored their impacts on the progression of prostate cancer.^9^, ^10^, ^11^ Therefore, the present study aims to investigate whether common genetic variants in anoctamin genes are associated with BCR‐free survival, which may help identify candidate genes and provide insight into the etiology of prostate cancer progression.

## 2 MATERIALS AND METHODS

### 2.1 Patient recruitment and data collection

In total, 641 patients were enrolled at three medical centers in Taiwan: Kaohsiung Medical University Hospital, Kaohsiung Veterans General Hospital, and National Taiwan University Hospital, as described previously.^12^ Biochemical recurrence was defined as two consecutive PSA measurements of 0.2 ng/mL or more after RP.^9^, ^13^, ^14^, ^15^ The institutional review board of Kaohsiung Medical University Hospital approved this study, and all participants provided written informed consent in accordance with the institutional guidelines.

### 2.2 Single‐nucleotide polymorphism selection and genotyping

We selected 213 single‐nucleotide polymorphisms (SNPs) from 10 anoctamin genes with a threshold of minor allele frequency (MAF) of >0.05 based on the 1000 Genomes data for Han Chinese in Beijing, China, and Southern Han Chinese.^16^ Single‐nucleotide polymorphism genotyping was conducted using Affymetrix Axiom Genotyping Arrays at the National Centre for Genome Medicine, Taiwan, as described previously.^17^ SNPs that deviated from Hardy‐Weinberg equilibrium (*P* < .005; N = 2) were removed, leaving a total of 211 SNPs for analyses.

### 2.3 Bioinformatics analysis

Bioinformatics and functional analyses were performed with multiple software tools and data sources: HaploReg^18^ for SNP functional prediction; lymphoblastoid cell data from the 1000 Genomes Project^16^ for expression quantitative trait loci (eQTL) analysis; and tumor gene expression data from the Memorial Sloan‐Kettering Cancer Center (MSKCC) Prostate Oncogenome^19^ and The Cancer Genome Atlas (TCGA)^20^ projects for gene expression survival analysis.

### 2.4 Statistical analysis

Statistical analyses were performed using Statistical Package for the Social Sciences software version 19.0.0 (IBM). A two‐sided *P* < .05 was considered to represent statistical significance; *q* values were calculated to reduce the probability of false positive findings.^21^

## 3 RESULTS

The basic characteristics of the 641 patients who underwent RP for localized prostate cancer are presented in Table 1. The median age of all patients was 66 years (interquartile range [IQR] 61.5‐70.0), and median PSA was 11.0 ng/mL (IQR 7.0‐18.4). Most patients had a Gleason score of 7‐10 (489, 76.3%), stage T1/T2 (361, 56.8%), and a negative surgical margin (457, 71.3%). Biochemical recurrence was observed in 226 (35.3%) patients during a median follow‐up of 51 months. Univariate Cox regression indicated that PSA, Gleason score, stage, and surgical margin were significantly associated with BCR (*P* < .001).

**Table 1:** Clinicopathologic characteristics of the study population

Characteristics	No BCR^a^	BCR^a^	*P*
No. of patients, N (%)	415 (64.7)	226 (35.3)	
Age at diagnosis			
Median, y (IQR)	66.0 (62.0‐70.0)	66.5 (61.0‐71.0)	.102
PSA at diagnosis, N (%)			
Median, ng/mL (IQR)	9.3 (6.2‐15.0)	14.8 (8.4‐26.3)	<.001
Pathologic Gleason score, N (%)			
2‐6	117 (77.0)	35 (23.0)	<.001
7‐10	298 (60.9)	191 (39.1)	
Pathologic stage, N (%)			
T1/T2	275 (76.2)	86 (23.8)	<.001
T3/T4/N1	139 (50.5)	136 (49.5)	
Surgical margin, N (%)			
Negative	320 (70.0)	137 (30.0)	<.001
Positive	95 (51.6)	89 (48.4)	

Note

Subtotals do not sum to 641 due to missing data.

Abbreviations: BCR, biochemical recurrence; IQR, interquartile range; PSA, prostate‐specific antigen.

a With a median follow‐up of 51 mo.

**Figure 1:**
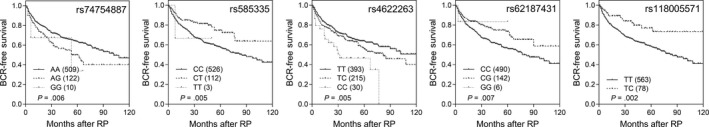
*ANO3* rs74754887, *ANO4* rs585335, *ANO5* rs4622263, *ANO7* rs62187431, and *ANO10* rs118005571 are associated with biochemical recurrence (BCR)‐free survival time. Values between brackets denote the number of patients

Cox regression analysis was performed to assess associations between a total of 211 common SNPs of 10 anoctamin genes and BCR (Table S1). Ten SNPs showed evidence of association at *P* < .05 and *q* = 0.232, indicating that 23.2% of the 10 SNPs were likely to be false positives. Therefore, seven SNPs with the lowest *P* (*ANO3* rs74754887, *ANO4* rs585335, *ANO4* rs1354228, *ANO5* rs4622263, *ANO7* rs62187431, *ANO7* rs76832527, and *ANO10* rs118005571) were considered noteworthy after multiple test correction. Since rs585335 and rs1354228 in *ANO4* and rs62187431 and rs76832527 in *ANO7* were in strong linkage disequilibrium (*r* ^2^ > .80), *ANO4* rs1354228 and *ANO7* rs76832527 were also excluded due to their lower significance, leaving five SNPs for further analyses (Table 2; Figure 1).

**Table 2:** SNPs associated with BCR in prostate cancer patients receiving RP

Gene SNP	N	BCR	5‐y BFS	HR (95% CI)	*P*	HR (95% CI)^a^	*P* a
Genotype							
*ANO3* rs74754887							
AA	509	170	63.5	1.00		1.00	
AG	122	52	50.0	1.45 (1.11‐1.90)	.007	1.33 (1.00‐1.76)	.050
GG	10	4	33.8				
*ANO4* rs585335							
CC	526	199	57.5	1.00		1.00	
CT	112	26	74.6	0.58 (0.39‐0.85)	.006	**0.63 (0.42‐0.95)**	**.025**
TT	3	1	66.7				
*ANO5* rs4622263							
TT	393	126	64.0	1.00		1.00	
TC	215	82	55.7	1.35 (1.09‐1.67)	.006	**1.44 (1.16‐1.79)**	**.001**
CC	30	17	46.6				
*ANO7* rs62187431							
CC	490	187	56.4	1.00		1.00	
CG	142	38	73.4	0.64 (0.45‐0.89)	.008	**0.67 (0.48‐0.94)**	**.022**
GG	6	1	83.3				
*ANO10* rs118005571							
TT	563	211	58.4	1.00		1.00	
TC	78	15	76.9	0.45 (0.26‐0.75)	.003	**0.44 (0.26‐0.75)**	**.002**

Note

Subtotals do not sum to 641 due to missing data.

*P* < .05 are in boldface.

Abbreviations: BCR, biochemical recurrence; BFS, BCR‐free survival; CI, confidence interval; HR, hazard ratio; RP, radical prostatectomy; SNP, single‐nucleotide polymorphism.

a Adjustment for age, PSA at diagnosis, pathologic Gleason score, stage, and surgical margin.

Multivariate Cox regression analysis was then performed to evaluate the robustness of these five SNPs on BCR with adjustment for known risk factors (Table 2). The risk of BCR significantly decreased with the number of *ANO4* rs585335 minor allele T, *ANO7* rs62187431 G, and *ANO10* rs118005571 C (*P* = .025, .022, and .002, respectively), but significantly increased with the number of *ANO5* rs4622263 C (*P* = .001).

**Figure 2:**
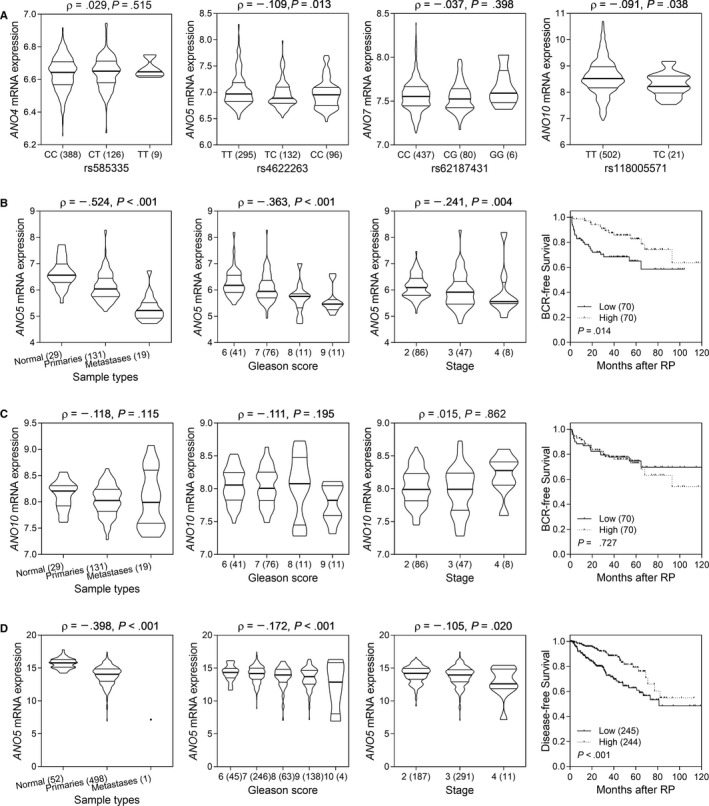
Functional analyses of candidate single‐nucleotide polymorphisms. A, Expression quantitative trait loci analyses identify two significant associations between rs4622263 and *ANO5*, and rs118005571 and *ANO10* in 523 HapMap lymphoblastoid cell lines. B, Lower expression of *ANO5* is correlated with prostate cancer, higher Gleason score, and stage, and shorter time to biochemical recurrence in Memorial Sloan‐Kettering Cancer Center cohort. C, Expression of *ANO10* is not altered over the course of prostate cancer progression. D, *ANO5* expression is consistently correlated with prostate cancer progression in the The Cancer Genome Atlas cohort. Values between brackets denote the number of patients. Rho: Spearman's rank correlation coefficient. RP, radical prostatectomy

We performed eQTL analysis using HapMap lymphoblastoid cell line data to test if these SNPs could influence gene expression. The minor allele C at rs4622263 was correlated with lower *ANO5* expression (*P* = .013; Figure 2A), and rs118005571 C was correlated with lower *ANO10* expression (*P* = .038). According to these results, we hypothesized that lower *ANO5* and higher *ANO10* expression would correlate with worse outcomes in prostate cancer. The associations between gene expression and prostate cancer outcomes were investigated in MSKCC and TCGA cohorts. Lower levels of *ANO5* expression were associated with more aggressive forms of prostate cancer, higher Gleason score, pathologic stage, and shorter time to BCR/disease‐free survival in both cohorts (Figure 2B,D). However, no significant associations were found between *ANO10* expression levels and prostate cancer (Figure 2C).

## 4 DISCUSSION

We conducted a comprehensive analysis of anoctamin gene SNPs with prostate cancer recurrence and found that *ANO5* rs4622263 was associated with unfavorable prognosis. This genotype‐outcome association was pronounced, even in the presence of known predictors of prostate cancer outcome. Moreover, rs4622263 influenced *ANO5* expression. These findings suggest that *ANO5* may have biological roles in prostate cancer and provide new insights into the mechanism of prostate cancer progression.

Anoctamins are a family of calcium‐activated ion channels and phospholipid scramblases.^22^ They have been shown to control compartmentalized calcium signaling by tethering endoplasmic reticulum (ER) calcium storage to the plasma membrane and promoting the release of calcium from ER via inositol trisphosphate and ryanodine receptors to regulate cell proliferation.^23^ Anoctamins can also function as phospholipid scramblases when activated by increases in calcium levels, whereby they promote cell death by moving phosphatidylserine from the inner to the outer leaflet of the plasma membrane.^24^ Further, the expression of several members of the anoctamin family, such as ANO1,^6^ ANO5,^25^ ANO7,^26^ and ANO9,^27^ is correlated with the development of malignant tumors. Expression of *ANO5* is downregulated in thyroid cancer, and knockdown of *ANO5* promotes cell migration and invasion via regulation of the JAK/STAT3 signaling pathway.^25^ However, the exact role of *ANO5* in prostate cancer has yet to be determined. The position of rs4622263 overlaps with a GATA binding protein 1‐bound region and is predicted to alter the regulatory motif transcription factor CP2‐like 1, which has been implicated in maintenance of pluripotency and self‐renewal in embryonic stem cells and cancers,^28^, ^29^ according to the experimental chromatin immunoprecipitation sequencing data in the HaploReg database. These data provide a possible explanation of the mechanisms underlying the association between *ANO5* rs4622263 and prostate cancer recurrence we observed.

A previous genome‐wide meta‐analysis of more than 25 000 men revealed no significant association between common genetic variants and prostate cancer‐specific survival.^11^ A possible reason might be the heterogeneous patient cohorts with different ethnic background and differences in patient care, which could dilute the effect of SNPs on prostate cancer‐specific survival. In contrast, another genome‐wide association study focused only on the northern European ancestry in Sweden and identified an association between the *AOX1* locus and prostate cancer‐specific survival.^10^ Therefore, the strengths of our study include a well‐characterized Taiwanese patient cohort from a defined geographical region, with complete medical information and significant follow‐up times, as well as a comprehensive coverage of common genetic variants across all genes of the anoctamin family.

There are several limitations to the present study. Firstly, our genetic association analyses were limited to a single multi‐center study. As our cohort was based on a Taiwanese patient population, it is not clear whether these results would apply in other ethnic groups. Although we used the *q*‐value for multiple test correction, it is still possible that some of our findings could be false discoveries. The limited sample size of the current study does not provide sufficient power to detect SNP associations with a low MAF; thus, additional cancer‐related genes might have been overlooked. Also, we have not determined the precise mechanisms for the effect of *ANO5* rs4622263 on prostate cancer progression, but functional annotation in HaploReg database provides clues to potential mechanisms, as described. Therefore, additional larger studies with multiethnic groups are needed to confirm our results, and further functional studies are also warranted to investigate the exact functions of rs4622263 or *ANO5* on prostate cancer progression.

In conclusion, the present study identified that rs4622263 is associated with BCR and may regulate *ANO5* gene expression. Lower expression of *ANO5* is correlated with worse BCR/disease‐free survival of prostate cancer. *ANO5* may impede the progression of prostate cancer, and rs4622263 could be a promising prognostic biomarker for personalized therapies.

## CONFLICT OF INTEREST

The authors have no conflict of interest to declare.

## Data Availability

The data that support the findings of this study are available from the corresponding author upon reasonable request.
