# Supplementary material for: Genetic association analysis identifies a role for ANO5 in prostate cancer progression
Source: Cancer Med. 2020 Feb 6;9(7):2372–8. doi: 10.1002/cam4.2909 (PMC7131841; doi:10.1002/cam4.2909)
Supplement: Supplementary file 1 [file CAM4-9-2372-s001.doc]

**Table S1.** Genotyped SNPs and the *P* values of their association with BCR after RP

| Gene | SNP ID | Chromosome | Position | HWE | MAF | Alleles | *P* | *q* |
| --- | --- | --- | --- | --- | --- | --- | --- | --- |
| *ANO1* | rs11233661 | 11 | 70089341 | 0.817 | 0.459 | A>G | 0.706 | 0.971 |
| *ANO1* | rs76643753 | 11 | 70089647 | 0.512 | 0.113 | G>A | 0.598 | 0.971 |
| *ANO1* | rs2515288 | 11 | 70098564 | 0.841 | 0.126 | C>T | 0.977 | 0.990 |
| *ANO1* | rs10898284 | 11 | 70100865 | 0.278 | 0.258 | G>A | 0.874 | 0.976 |
| *ANO1* | rs2509147 | 11 | 70103221 | 0.033 | 0.347 | C>T | 0.431 | 0.971 |
| *ANO1* | rs10751121 | 11 | 70105398 | 0.286 | 0.072 | G>A | 0.634 | 0.971 |
| *ANO1* | rs732849 | 11 | 70106987 | 0.399 | 0.347 | T>C | 0.791 | 0.971 |
| *ANO1* | rs1893086 | 11 | 70109193 | 0.727 | 0.160 | T>G | 0.714 | 0.971 |
| *ANO1* | rs2509185 | 11 | 70110902 | 0.230 | 0.161 | A>G | 0.528 | 0.971 |
| *ANO1* | rs11234403 | 11 | 70111614 | 0.568 | 0.084 | G>T | 0.843 | 0.971 |
| *ANO1* | rs2509142 | 11 | 70117968 | 0.006 | 0.458 | C>T | 0.795 | 0.971 |
| *ANO1* | rs2515267 | 11 | 70130087 | 0.249 | 0.450 | G>A | 0.697 | 0.971 |
| *ANO1* | rs2509177 | 11 | 70133454 | 0.183 | 0.077 | A>G | 0.737 | 0.971 |
| *ANO1* | rs74485435 | 11 | 70144548 | 0.541 | 0.053 | G>A | 0.200 | 0.817 |
| *ANO1* | rs77701718 | 11 | 70152604 | 0.287 | 0.084 | C>T | 0.864 | 0.971 |
| *ANO1* | rs3781663 | 11 | 70153146 | 0.113 | 0.242 | A>G | 0.750 | 0.971 |
| *ANO1* | rs10898827 | 11 | 70173307 | 0.533 | 0.189 | G>A | 0.367 | 0.971 |
| *ANO1* | rs7127129 | 11 | 70181807 | 0.623 | 0.481 | G>A | 0.968 | 0.990 |
| *ANO2* | rs11608648 | 12 | 5533754 | 0.751 | 0.166 | G>A | 0.179 | 0.817 |
| *ANO2* | rs11063742 | 12 | 5538821 | 0.921 | 0.053 | T>C | 0.997 | 0.997 |
| *ANO2* | rs17181291 | 12 | 5551559 | 0.740 | 0.345 | G>A | 0.519 | 0.971 |
| *ANO2* | rs61908059 | 12 | 5568422 | 1.000 | 0.125 | G>A | 0.795 | 0.971 |
| *ANO2* | rs58399805 | 12 | 5570938 | 0.727 | 0.093 | T>A | 0.366 | 0.971 |
| *ANO2* | rs11063761 | 12 | 5571758 | 0.452 | 0.147 | G>A | 0.326 | 0.971 |
| *ANO2* | rs7133109 | 12 | 5579217 | 0.147 | 0.175 | T>G | 0.767 | 0.971 |
| *ANO2* | rs3886663 | 12 | 5602745 | 0.851 | 0.437 | A>G | 0.122 | 0.694 |
| *ANO2* | rs7969805 | 12 | 5603375 | 0.704 | 0.167 | T>C | 0.599 | 0.971 |
| *ANO2* | rs3782598 | 12 | 5616489 | 0.885 | 0.241 | G>A | 0.095 | 0.645 |
| *ANO2* | rs3782600 | 12 | 5626643 | 0.468 | 0.158 | T>C | 0.518 | 0.971 |
| *ANO2* | rs9943746 | 12 | 5637126 | 0.659 | 0.317 | C>T | 0.492 | 0.971 |
| *ANO2* | rs393861 | 12 | 5639881 | 0.978 | 0.371 | G>A | 0.865 | 0.971 |
| *ANO2* | rs11063791 | 12 | 5640002 | 1.000 | 0.196 | C>T | 0.162 | 0.795 |
| *ANO2* | rs386440 | 12 | 5640115 | 0.162 | 0.251 | T>A | 0.828 | 0.971 |
| *ANO2* | rs2110166 | 12 | 5642176 | 0.771 | 0.124 | C>T | 0.512 | 0.971 |
| *ANO2* | rs381373 | 12 | 5646161 | 0.642 | 0.405 | T>C | 0.949 | 0.990 |
| *ANO2* | rs77590605 | 12 | 5660659 | 0.630 | 0.085 | G>A | 0.412 | 0.971 |
| *ANO2* | rs759518 | 12 | 5662555 | 0.299 | 0.307 | C>T | 0.633 | 0.971 |
| *ANO2* | rs2215736 | 12 | 5678456 | 0.553 | 0.402 | T>C | 0.693 | 0.971 |
| *ANO2* | rs12579350 | 12 | 5687935 | 0.648 | 0.117 | G>A | 0.705 | 0.971 |
| *ANO2* | rs7965813 | 12 | 5690218 | 0.744 | 0.396 | G>T | 0.536 | 0.971 |
| *ANO2* | rs16933825 | 12 | 5733725 | 1.000 | 0.062 | T>C | 0.281 | 0.944 |
| *ANO2* | rs10849329 | 12 | 5740406 | 0.373 | 0.384 | C>T | 0.085 | 0.645 |
| *ANO2* | rs117460555 | 12 | 5740701 | 0.956 | 0.052 | A>G | 0.710 | 0.971 |
| *ANO2* | rs17195529 | 12 | 5749295 | 0.270 | 0.161 | A>G | 0.209 | 0.817 |
| *ANO2* | rs10459134 | 12 | 5770685 | 0.302 | 0.168 | C>T | 0.066 | 0.645 |
| *ANO2* | rs1860958 | 12 | 5781006 | 1.000 | 0.074 | C>T | 0.634 | 0.971 |
| *ANO2* | rs1807278 | 12 | 5790806 | 0.169 | 0.240 | T>C | 0.458 | 0.971 |
| *ANO2* | rs7301894 | 12 | 5809384 | 0.903 | 0.464 | G>A | 0.485 | 0.971 |
| *ANO2* | rs11832095 | 12 | 5814201 | 0.776 | 0.276 | G>A | 0.205 | 0.817 |
| *ANO2* | rs74899368 | 12 | 5814231 | 0.615 | 0.191 | G>A | 0.848 | 0.971 |
| *ANO2* | rs76082116 | 12 | 5839732 | 0.092 | 0.251 | T>C | 0.484 | 0.971 |
| *ANO2* | rs116896439 | 12 | 5846326 | 1.000 | 0.075 | C>T | 0.603 | 0.971 |
| *ANO2* | rs77016945 | 12 | 5873103 | 1.000 | 0.069 | T>C | 0.906 | 0.990 |
| *ANO2* | rs7973953 | 12 | 5875268 | 0.435 | 0.456 | T>C | 0.796 | 0.971 |
| *ANO2* | rs75419846 | 12 | 5881109 | 0.070 | 0.307 | C>T | 0.672 | 0.971 |
| *ANO2* | rs7486935 | 12 | 5901078 | 0.385 | 0.235 | A>G | 0.408 | 0.971 |
| *ANO2* | rs73257264 | 12 | 5910700 | 0.358 | 0.300 | A>G | 0.545 | 0.971 |
| *ANO2* | rs17786442 | 12 | 5920294 | 0.639 | 0.184 | C>T | 0.449 | 0.971 |
| *ANO2* | rs7398532 | 12 | 5942443 | 0.410 | 0.227 | A>G | 0.086 | 0.645 |
| *ANO2* | rs6489680 | 12 | 5945229 | 0.805 | 0.150 | G>A | 0.276 | 0.944 |
| *ANO3* | rs12802779 | 11 | 26203339 | 0.898 | 0.176 | C>T | 0.731 | 0.971 |
| *ANO3* | rs35766300 | 11 | 26221150 | 0.521 | 0.176 | G>A | 0.667 | 0.971 |
| *ANO3* | rs3101360 | 11 | 26234444 | 0.118 | 0.277 | T>C | 0.110 | 0.645 |
| *ANO3* | rs12161755 | 11 | 26235043 | 0.021 | 0.180 | T>A | 0.422 | 0.971 |
| *ANO3* | rs9332433 | 11 | 26241538 | 0.608 | 0.147 | T>C | 0.719 | 0.971 |
| *ANO3* | rs11029419 | 11 | 26243400 | 0.348 | 0.161 | T>G | 0.820 | 0.971 |
| *ANO3* | rs11029428 | 11 | 26254107 | 0.356 | 0.161 | T>C | 0.476 | 0.971 |
| *ANO3* | rs11029447 | 11 | 26259756 | 0.533 | 0.145 | A>G | 0.644 | 0.971 |
| *ANO3* | rs80151205 | 11 | 26260831 | 0.287 | 0.153 | C>T | 0.588 | 0.971 |
| *ANO3* | rs12419504 | 11 | 26271055 | 0.982 | 0.134 | T>A | 0.040 | 0.567 |
| *ANO3* | rs80328008 | 11 | 26271574 | 0.646 | 0.118 | T>A | 0.489 | 0.971 |
| *ANO3* | rs35624765 | 11 | 26283483 | 0.491 | 0.152 | G>A | 0.649 | 0.971 |
| *ANO3* | rs11029505 | 11 | 26301188 | 0.239 | 0.157 | A>C | 0.525 | 0.971 |
| *ANO3* | rs11029518 | 11 | 26323860 | 0.404 | 0.154 | A>G | 0.863 | 0.971 |
| *ANO3* | rs10834964 | 11 | 26340027 | 0.780 | 0.311 | T>G | 0.108 | 0.645 |
| *ANO3* | rs11605942 | 11 | 26354559 | 0.585 | 0.185 | T>G | 0.662 | 0.971 |
| *ANO3* | rs10834971 | 11 | 26359258 | 0.385 | 0.187 | C>A | 0.046 | 0.571 |
| *ANO3* | rs117551578 | 11 | 26374622 | 0.089 | 0.067 | A>G | 0.318 | 0.971 |
| *ANO3* | rs7110540 | 11 | 26376125 | 0.630 | 0.336 | T>C | 0.056 | 0.622 |
| *ANO3* | rs1563956 | 11 | 26379832 | 0.742 | 0.354 | A>G | 0.027 | 0.475 |
| *ANO3* | rs12287530 | 11 | 26387869 | 0.428 | 0.318 | G>A | 0.102 | 0.645 |
| *ANO3* | rs67498125 | 11 | 26388409 | 0.302 | 0.200 | C>T | 0.886 | 0.983 |
| *ANO3* | rs74754887 | 11 | 26414963 | 0.469 | 0.110 | A>G | 0.007 | **0.232** |
| *ANO3* | rs16915651 | 11 | 26418163 | 0.353 | 0.200 | A>C | 0.794 | 0.971 |
| *ANO3* | rs10742138 | 11 | 26428345 | 0.978 | 0.307 | T>C | 0.319 | 0.971 |
| *ANO3* | rs10047414 | 11 | 26444733 | 0.933 | 0.308 | G>A | 0.145 | 0.728 |
| *ANO3* | rs10834982 | 11 | 26457387 | 0.650 | 0.325 | G>A | 0.280 | 0.944 |
| *ANO3* | rs76962336 | 11 | 26473440 | 1.000 | 0.066 | T>C | 0.361 | 0.971 |
| *ANO3* | rs1125621 | 11 | 26501246 | 0.208 | 0.352 | C>T | 0.105 | 0.645 |
| *ANO3* | rs10834997 | 11 | 26505401 | 0.236 | 0.253 | A>G | 0.560 | 0.971 |
| *ANO3* | rs368786 | 11 | 26522943 | 0.274 | 0.179 | A>G | 0.332 | 0.971 |
| *ANO3* | rs375562 | 11 | 26537326 | 0.366 | 0.050 | T>C | 0.830 | 0.971 |
| *ANO3* | rs3802751 | 11 | 26537400 | 0.530 | 0.063 | T>C | 0.930 | 0.990 |
| *ANO3* | rs367336 | 11 | 26538420 | 0.181 | 0.183 | T>G | 0.204 | 0.817 |
| *ANO3* | rs448290 | 11 | 26551048 | 0.073 | 0.200 | A>G | 0.382 | 0.971 |
| *ANO3* | rs12271124 | 11 | 26563891 | 0.848 | 0.350 | C>T | 0.654 | 0.971 |
| *ANO3* | rs293980 | 11 | 26565586 | 0.553 | 0.446 | T>C | 0.986 | 0.991 |
| *ANO3* | rs2063278 | 11 | 26570430 | 0.911 | 0.406 | A>G | 0.241 | 0.892 |
| *ANO3* | rs73434317 | 11 | 26577158 | 0.041 | 0.234 | T>C | 0.303 | 0.971 |
| *ANO3* | rs364370 | 11 | 26582323 | 1.000 | 0.164 | C>T | 0.637 | 0.971 |
| *ANO3* | rs12295638 | 11 | 26583784 | 0.079 | 0.362 | T>C | 0.560 | 0.971 |
| *ANO3* | rs4923366 | 11 | 26598221 | 0.466 | 0.144 | G>A | 0.658 | 0.971 |
| *ANO3* | rs11603582 | 11 | 26600936 | 0.434 | 0.342 | T>C | 0.469 | 0.971 |
| *ANO3* | rs293956 | 11 | 26601939 | 0.740 | 0.081 | G>A | 0.826 | 0.971 |
| *ANO3* | rs439961 | 11 | 26618827 | 0.169 | 0.052 | C>T | 0.567 | 0.971 |
| *ANO3* | rs11029650 | 11 | 26622088 | 0.306 | 0.351 | T>C | 0.087 | 0.645 |
| *ANO3* | rs7110072 | 11 | 26640143 | 0.149 | 0.079 | C>T | 0.714 | 0.971 |
| *ANO3* | rs10734380 | 11 | 26647548 | 0.568 | 0.299 | A>G | 0.754 | 0.971 |
| *ANO3* | rs34892008 | 11 | 26654754 | 0.984 | 0.302 | C>T | 0.139 | 0.715 |
| *ANO3* | rs7942403 | 11 | 26663579 | 0.480 | 0.457 | A>G | 0.180 | 0.817 |
| *ANO4* | rs10860627 | 12 | 100735506 | 0.885 | 0.457 | T>C | 0.451 | 0.971 |
| *ANO4* | rs4764751 | 12 | 100740134 | 0.376 | 0.171 | A>G | 0.954 | 0.990 |
| *ANO4* | rs11110507 | 12 | 100754438 | 0.877 | 0.224 | G>A | 0.573 | 0.971 |
| *ANO4* | rs1399456 | 12 | 100761858 | 0.998 | 0.487 | G>A | 0.802 | 0.971 |
| *ANO4* | rs1606761 | 12 | 100771591 | 0.241 | 0.206 | C>T | 0.579 | 0.971 |
| *ANO4* | rs75788061 | 12 | 100774801 | 0.795 | 0.069 | C>G | 0.965 | 0.990 |
| *ANO4* | rs60331076 | 12 | 100796149 | 0.422 | 0.382 | C>T | 0.185 | 0.817 |
| *ANO4* | rs75112059 | 12 | 100800929 | 0.089 | 0.215 | C>T | 0.543 | 0.971 |
| *ANO4* | rs7966442 | 12 | 100820335 | 0.552 | 0.220 | C>T | 0.645 | 0.971 |
| *ANO4* | rs79780223 | 12 | 100821850 | 0.738 | 0.230 | G>A | 0.979 | 0.990 |
| *ANO4* | rs1399439 | 12 | 100827461 | 0.301 | 0.071 | A>G | 0.737 | 0.971 |
| *ANO4* | rs11110553 | 12 | 100845393 | 0.699 | 0.209 | G>T | 0.209 | 0.817 |
| *ANO4* | rs10778081 | 12 | 100847185 | 0.241 | 0.189 | C>T | 0.039 | 0.567 |
| *ANO4* | rs10507121 | 12 | 100857776 | 0.715 | 0.088 | G>T | 0.337 | 0.971 |
| *ANO4* | rs74907998 | 12 | 100864435 | 1.000 | 0.074 | T>G | 0.813 | 0.971 |
| *ANO4* | rs76788350 | 12 | 100880580 | 0.531 | 0.076 | C>T | 0.456 | 0.971 |
| *ANO4* | rs2672502 | 12 | 100896606 | 0.341 | 0.093 | C>T | 0.011 | **0.232** |
| *ANO4* | rs1354228 | 12 | 100907319 | 0.364 | 0.092 | G>T | 0.008 | **0.232** |
| *ANO4* | rs602734 | 12 | 100925956 | 0.091 | 0.468 | T>C | 0.076 | 0.645 |
| *ANO4* | rs657331 | 12 | 100927628 | 0.318 | 0.474 | G>A | 0.188 | 0.817 |
| *ANO4* | rs585335 | 12 | 100944217 | 0.386 | 0.091 | C>T | 0.006 | **0.232** |
| *ANO4* | rs12821670 | 12 | 100959546 | 0.803 | 0.460 | C>T | 0.909 | 0.990 |
| *ANO4* | rs11110605 | 12 | 100971519 | 0.860 | 0.219 | G>A | 0.964 | 0.990 |
| *ANO4* | rs4764631 | 12 | 100978084 | 0.660 | 0.222 | A>G | 0.837 | 0.971 |
| *ANO4* | rs609728 | 12 | 100981868 | 0.037 | 0.140 | C>T | 0.327 | 0.971 |
| *ANO4* | rs17485225 | 12 | 101006921 | 0.174 | 0.133 | G>A | 0.482 | 0.971 |
| *ANO4* | rs17406903 | 12 | 101007975 | 0.610 | 0.118 | C>A | 0.097 | 0.645 |
| *ANO4* | rs560441 | 12 | 101023771 | 0.333 | 0.266 | G>A | 0.282 | 0.944 |
| *ANO4* | rs2653447 | 12 | 101050865 | 0.413 | 0.429 | T>G | 0.009 | **0.232** |
| *ANO4* | rs7956620 | 12 | 101053863 | 0.149 | 0.177 | A>G | 0.731 | 0.971 |
| *ANO4* | rs73156636 | 12 | 101064274 | 0.310 | 0.067 | A>G | 0.919 | 0.990 |
| *ANO4* | rs629585 | 12 | 101069513 | 0.534 | 0.112 | A>G | 0.890 | 0.983 |
| *ANO4* | rs147770411 | 12 | 101082712 | 1.000 | 0.091 | A>G | 0.440 | 0.971 |
| *ANO4* | rs589657 | 12 | 101088077 | 0.922 | 0.456 | A>G | 0.448 | 0.971 |
| *ANO4* | rs677871 | 12 | 101093994 | 0.960 | 0.455 | T>C | 0.543 | 0.971 |
| *ANO4* | rs1055734 | 12 | 101126911 | 0.719 | 0.171 | A>G | 0.469 | 0.971 |
| *ANO5* | rs10833635 | 11 | 21808818 | 0.396 | 0.120 | A>G | 0.962 | 0.990 |
| *ANO5* | rs76033441 | 11 | 21830484 | 1.000 | 0.050 | G>A | 0.804 | 0.971 |
| *ANO5* | rs1032229 | 11 | 21831623 | 0.160 | 0.443 | C>T | 0.708 | 0.971 |
| *ANO5* | rs2029301 | 11 | 21838620 | 0.257 | 0.110 | A>G | 0.695 | 0.971 |
| *ANO5* | rs7122488 | 11 | 21852707 | 0.034 | 0.344 | C>T | 0.774 | 0.971 |
| *ANO5* | rs4922945 | 11 | 21873268 | 0.517 | 0.098 | G>A | 0.586 | 0.971 |
| *ANO5* | rs11026318 | 11 | 21875334 | 0.481 | 0.184 | C>T | 0.195 | 0.817 |
| *ANO5* | rs9787783 | 11 | 21894475 | 0.030 | 0.264 | A>G | 0.268 | 0.944 |
| *ANO5* | rs17233735 | 11 | 21905060 | 1.000 | 0.102 | A>G | 0.017 | 0.326 |
| *ANO5* | rs7112564 | 11 | 21929555 | 0.805 | 0.490 | A>G | 0.132 | 0.714 |
| *ANO5* | rs11026343 | 11 | 21947386 | 0.590 | 0.358 | T>C | 0.086 | 0.645 |
| *ANO5* | rs324228 | 11 | 21966015 | 0.726 | 0.477 | G>T | 0.106 | 0.645 |
| *ANO5* | rs12417691 | 11 | 21994136 | 0.728 | 0.140 | A>G | 0.096 | 0.645 |
| *ANO5* | rs11026373 | 11 | 22005113 | 0.796 | 0.097 | A>G | 0.391 | 0.971 |
| *ANO5* | rs10766907 | 11 | 22012592 | 1.000 | 0.055 | C>T | 0.704 | 0.971 |
| *ANO5* | rs11026387 | 11 | 22032878 | 0.954 | 0.258 | T>C | 0.474 | 0.971 |
| *ANO5* | rs10833698 | 11 | 22064400 | 1.000 | 0.060 | T>C | 0.477 | 0.971 |
| *ANO5* | rs11026407 | 11 | 22083484 | 0.816 | 0.343 | A>C | 0.291 | 0.959 |
| *ANO5* | rs11026412 | 11 | 22092547 | 0.383 | 0.368 | G>A | 0.666 | 0.971 |
| *ANO5* | rs4567457 | 11 | 22116565 | 0.664 | 0.168 | C>A | 0.408 | 0.971 |
| *ANO5* | rs4622263 | 11 | 22151826 | 1.000 | 0.216 | T>C | 0.006 | **0.232** |
| *ANO5* | rs34291608 | 11 | 22163166 | 1.000 | 0.207 | T>G | 0.052 | 0.610 |
| *ANO5* | rs11026452 | 11 | 22185949 | 0.746 | 0.219 | C>A | 0.408 | 0.971 |
| *ANO5* | rs4414211 | 11 | 22223237 | 0.699 | 0.357 | C>T | 0.042 | 0.567 |
| *ANO5* | rs7115712 | 11 | 22243727 | 1.000 | 0.205 | G>A | 0.098 | 0.645 |
| *ANO5* | rs192732414 | 11 | 22250515 | 1.000 | 0.069 | A>G | 0.977 | 0.990 |
| *ANO5* | rs10766930 | 11 | 22280261 | 0.976 | 0.338 | A>G | 0.196 | 0.817 |
| *ANO6* | rs117017504 | 12 | 45229883 | 0.204 | 0.058 | G>A | 0.815 | 0.971 |
| *ANO6* | rs12815334 | 12 | 45263024 | 0.523 | 0.351 | G>T | 0.427 | 0.971 |
| *ANO6* | rs79980430 | 12 | 45280320 | 0.077 | 0.122 | A>G | 0.638 | 0.971 |
| *ANO6* | rs1913692 | 12 | 45308641 | 1.000 | 0.444 | A>G | 0.352 | 0.971 |
| *ANO6* | rs4768606 | 12 | 45314160 | 0.095 | 0.067 | T>C | 0.580 | 0.971 |
| *ANO6* | rs10785571 | 12 | 45332832 | 0.659 | 0.094 | T>G | 0.541 | 0.971 |
| *ANO6* | rs117914094 | 12 | 45347231 | 0.834 | 0.060 | G>T | 0.979 | 0.990 |
| *ANO6* | rs17095830 | 12 | 45381125 | 0.722 | 0.100 | A>G | 0.842 | 0.971 |
| *ANO6* | rs11183023 | 12 | 45423855 | 0.051 | 0.343 | G>T | 0.276 | 0.944 |
| *ANO7* | rs2302054 | 2 | 241190100 | 0.356 | 0.481 | G>A | 0.487 | 0.971 |
| *ANO7* | rs11676246 | 2 | 241194877 | 0.430 | 0.148 | T>C | 0.966 | 0.990 |
| *ANO7* | rs4675824 | 2 | 241198919 | 0.346 | 0.102 | T>C | 0.071 | 0.645 |
| *ANO7* | rs2013250 | 2 | 241200076 | 0.426 | 0.148 | C>T | 0.970 | 0.990 |
| *ANO7* | rs2074840 | 2 | 241202304 | 0.636 | 0.056 | C>T | 0.852 | 0.971 |
| *ANO7* | rs9679681 | 2 | 241207849 | 1.000 | 0.144 | C>A | 0.681 | 0.971 |
| *ANO7* | rs10192730 | 2 | 241209658 | 0.173 | 0.453 | G>A | 0.125 | 0.694 |
| *ANO7* | rs55998353 | 2 | 241211673 | 0.326 | 0.070 | C>T | 0.482 | 0.971 |
| *ANO7* | rs62187431 | 2 | 241212376 | 0.277 | 0.121 | C>G | 0.008 | **0.232** |
| *ANO7* | rs749264 | 2 | 241215892 | 1.000 | 0.099 | C>A | 0.644 | 0.971 |
| *ANO7* | rs76832527 | 2 | 241217826 | 0.765 | 0.121 | G>A | 0.008 | **0.232** |
| *ANO7* | rs62186361 | 2 | 241229103 | 0.460 | 0.107 | G>A | 0.010 | **0.232** |
| *ANO7* | rs78113223 | 2 | 241236211 | 0.180 | 0.060 | C>T | 0.655 | 0.971 |
| *ANO8* | rs8102944 | 19 | 17327833 | 0.286 | 0.276 | A>G | 0.762 | 0.971 |
| *ANO9* | rs78972632 | 11 | 431757 | 0.604 | 0.125 | C>T | 0.690 | 0.971 |
| *ANO9* | rs74974272 | 11 | 434540 | 0.969 | 0.093 | G>A | 0.499 | 0.971 |
| *ANO9* | rs7481614 | 11 | 438662 | 0.163 | 0.091 | G>A | 0.731 | 0.971 |
| *ANO10* | rs150764868 | 3 | 43364906 | 0.643 | 0.077 | G>A | 0.981 | 0.990 |
| *ANO10* | rs6441772 | 3 | 43380948 | 0.476 | 0.063 | T>C | 0.839 | 0.971 |
| *ANO10* | rs34557672 | 3 | 43393160 | 0.688 | 0.092 | C>A | 0.234 | 0.882 |
| *ANO10* | rs78131568 | 3 | 43413692 | 0.168 | 0.060 | T>C | 0.718 | 0.971 |
| *ANO10* | rs78791951 | 3 | 43426111 | 0.668 | 0.108 | C>T | 0.813 | 0.971 |
| *ANO10* | rs12492694 | 3 | 43429642 | 0.436 | 0.223 | A>C | 0.215 | 0.825 |
| *ANO10* | rs117035952 | 3 | 43517972 | 0.145 | 0.128 | C>T | 0.139 | 0.715 |
| *ANO10* | rs12638282 | 3 | 43549650 | 0.549 | 0.180 | A>G | 0.043 | 0.567 |
| *ANO10* | rs118005571 | 3 | 43564465 | 0.158 | 0.061 | T>C | 0.003 | **0.232** |
| *ANO10* | rs17030517 | 3 | 43622546 | 0.789 | 0.118 | G>A | 0.069 | 0.645 |
| *ANO10* | rs72622910 | 3 | 43672207 | 1.000 | 0.107 | G>A | 0.943 | 0.990 |
| *ANO10* | rs72622914 | 3 | 43690029 | 1.000 | 0.131 | A>C | 0.487 | 0.971 |

Abbreviations: SNP, single nucleotide polymorphism; BCR, biochemical recurrence; RP, radical prostatectomy; MAF, minor alleles frequency; HWE, Hardy-Weinberg equilibrium.

*q* < 0.300 is in boldface.
